# The Will to Recover

**Published:** 1916-02-05

**Authors:** 


### The Hospital

The Workers' Newspaper of Administrative Medicine and Institutional Life, Administration, National Insurance and Health.

No. 1547, Vol. LIX. SATURDAY, FEBRUARY 5, 1916. PRICE ONE PENNY.

### THE WILL TO RECOVER.

Among the therapeutic agencies which are generally credited with the capacity to influence favourably the course and issue of bodily illness, a place is usually found for the mental resolution of the patient. The factor, it is allowed, cannot be stated in terms of measurement, and indeed anything like precise definition of it is not possible; but that it exists and operates is widely acknowledged. The acknowledgment, too, comes both from physicians, to whom the phenomena and treatment of disease afford opportunities for scientific study and expert craftsmanship, and also from lay observers or from the sufferers themselves. For various reasons the healing influences of mental states have been pushed into prominence of recent years, and though the discussions and practices associated with this topic have not always been edifying, this does not justify a summary dismissal of the subject as unworthy of consideration. In any event, the fact remains that there is substantial testimony to the value of resolution and hopefulness as aids to recovery, and hence it is reasonable to urge that the study of these agencies for practical therapeutic purposes ought to be encouraged and systematised.

It happens that the value of resolution and determination as factors bearing on endurance and action is just now very prominent in the public eye. The will to conquer is advocated in all the belligerent nations, and it is advocated in the belief that its operation will be effective in the world of practical affairs. Not merely to overcome the anxieties of the home populations, but also to strengthen the arm of the combatant, is the aim of the national leaders when they appeal to the mental resolution of their countrymen. It is on the same principle that the military commander stimulates his troops as they advance to meet the foe. He knows by experience that appropriate words—or rather that the mental picture these convey—will arouse a degree of capacity which might otherwise have remained latent. A more homely illustration of the same principle is seen in the employment of a "pacemaker" by those who are in training for some great effort, or by a person endeavouring to break the time record of some particular racing achievement. The mental condition induced by a consciousness of companionship or of competition helps the individual to call upon his store of physiological reserves and to bring these into fruitful and effective operation. These exist, but something is necessary to secure their display, and this something is provided by a mental stimulus. For many this stimulus must be supplied by external circumstances or by the influence of a second and sympathetic personality. But the exceptional individual finds what is necessary in his own resources.

It is, we venture to say, on the lines here defined that is to be found the rational explanation of instances in which patients have successfully defied the gloomy prophecies which their symptoms imposed. Some reserves of capacity for resistance and endurance have been summoned into action under the influence either of outside agencies or by the unconquerable will of the patient himself; and the triumphs which are recounted as miracles by lovers of the dramatic have really a comparatively simple physiological explanation. In the field of functional disease, where in consequence of inhibited brain action such conditions as blindness, deafness, or paralysis exist, "marvellous" recoveries under mental stimulation are common enough, and the war has furnished many of these as texts for newspaper paragraphs in quarters where the nine days' wonder is always welcome. They lend themselves to sensational treatment, and this they often receive. But in principle there may be found parallel experiences even when gross organic disease is present, and this both of the acute and the chronic form. Not, of course, that any measure of mental resolve can achieve a permanent victory, for the dread victor awaits us all; nor that determination can restore organs damaged beyond repair. But the fight may be prolonged by the resolution to continue the struggle and to endure even until all the latent capacities of flesh and blood have gained their final expression; and even then, as with the dying gladiator, there may be possible the "manly brow" which "consents to death but conquers agony." The man who turns his face to the wall yields to the influences which oppress him without this last degree of resistance, while the sterner soul makes the one further effort which may perchance carry him into safety or, short of this, may at least secure him some temporary respite.

In thus seeking to apply in the world of therapeutics a truth which is widely recognised in other areas there are practical applications which relate both to the physician and to the patient. The former must not omit from his armamentarium agencies which are very real though he may not find them easy of definition. As for their mode of action they are not so mysterious as many would suggest, and in any event it is their efficacy which is the prime consideration. Every experienced physician knows how rarely can an inevitably fatal prognosis as to the immediate future be justified, and unless he is an exceptionally prudent man time and the event must have often contradicted his forebodings. Hence, it is his plain duty to help, by the stimulus of hopefulness and courage, the resolve and endurance which may mean all the difference between victory and defeat. Conquered in the end he many times must be, but let it not be without a full effort for success. For the patient obviously there is a similar duty. Possibly he may possess the iron resolution which knows no yielding, and if so, such chance as exists is in his hands. But this virtue is not the gift of a moment. And hence out of the considerations above suggested arises the question of early training and discipline. For the qualities which fit a man to meet the disaster and strain of a sick-bed and to struggle to victory will exist on the one hand, or will not exist on the other, according as they have been cultivated or neglected in earlier years. There is thus, apart from other excellencies, therapeutic value in the education which makes youth "strong in will—to strive, to seek, to find and not to yield." The will to conquer may find its therapeutic expression either in the crisis of acute disease or in the prolonged strain of a debilitating illness. But if it is to serve in the emergency it must be cultivated in advance.

